# Impact of collaborative learning on Internet knowledge learning performances and experiences of older adults

**DOI:** 10.1093/geroni/igaf122.2947

**Published:** 2025-12-31

**Authors:** Zhiyu Fan, Yinong An, Huamao Peng

**Affiliations:** Beijing Normal University, Beijing, China (People’s Republic); Beijing Normal University, Beijing, China (People’s Republic); Beijing Normal University, Beijing, China (People’s Republic)

## Abstract

Lifelong learning is an important part of active aging. Collaborative learning, characterized by learner-centered autonomy and social interaction, is generally considered more effective than individual learning. However, its applicability to older adults remains unclear due to their distinct learning characteristics. Cognitive interactions in collaboration may deepen learning but could also impose additional cognitive burdens, while socio-emotional interactions may foster engagement and mitigate cognitive aging. This study examined the effect of collaborative learning on older adults’ Internet knowledge learning outcomes and experiences, and examined the role of different interactions in the process. Study 1 (n = 167) employed a 3 × 2 mixed design, with learning approach (individual/ dyads collaborative/ triads collaborative) as between-subject variable and test (pre/ post) as within-subject variable. While no significant differences in learning performance were observed across conditions, the dyads reported more positive learning experiences (higher satisfaction and greater willingness for continued learning). Study 2 (n = 76) utilized a 2 × 2 mixed design, with collaborative learning approach (cognitive interactions guided dyads/ socio-emotional interactions guided dyads) as between-subject variable and test (pre/ post) as within-subject variable. Study 2 incorporated data from Study 1’s individual and unstructured dyadic conditions. Results replicated Study 1’s findings, showing no performance differences. However, dyads without structured guidance (in Study 1) reported more positive learning experiences (higher learning satisfaction). These findings suggest that unstructured dyadic collaboration offers a balanced approach for older adults, maintaining learning performance while enhancing learning experiences. This approach appears particularly suitable for Internet knowledge acquisition in older populations, providing a supportive yet flexible learning environment.

